# Nurse-Led Intervention to Improve Knowledge of Medications in Survivors of Stroke or Transient Ischemic Attack: A Cluster Randomized Controlled Trial

**DOI:** 10.3389/fneur.2016.00205

**Published:** 2016-11-18

**Authors:** Muideen T. Olaiya, Dominique A. Cadilhac, Joosup Kim, David Ung, Mark R. Nelson, Velandai K. Srikanth, Christopher F. Bladin, Richard P. Gerraty, Sharyn M. Fitzgerald, Thanh G. Phan, Judith Frayne, Amanda G. Thrift

**Affiliations:** ^1^Stroke and Ageing Research Centre, School of Clinical Sciences at Monash Health, Monash University, Clayton, VIC, Australia; ^2^Stroke Division, The Florey Institute of Neuroscience and Mental Health, Heidelberg, VIC, Australia; ^3^Menzies Institute for Medical Research, Hobart, TAS, Australia; ^4^Department of Epidemiology and Preventive Medicine, Monash University, Melbourne, VIC, Australia; ^5^Department of Neurosciences, Box Hill Hospital, Box Hill, VIC, Australia; ^6^Department of Medicine, Epworth Healthcare, Richmond, VIC, Australia; ^7^Department of Neurology, Alfred Hospital, Prahan, VIC, Australia

**Keywords:** randomized controlled trial, stroke, nursing intervention, patient medication knowledge, secondary prevention

## Abstract

**Introduction:**

Limited evidence exists on effective interventions to improve knowledge of preventive medications in patients with chronic diseases, such as stroke. We investigated the effectiveness of a nurse-led intervention, where a component was to improve knowledge of prevention medications, in patients with stroke or transient ischemic attack (TIA).

**Methods:**

Prospective sub-study of the Shared Team Approach between Nurses and Doctors for Improved Risk Factor Management, a randomized controlled trial of risk factor management. We recruited patients aged ≥18 years and hospitalized for stroke/TIA. The intervention comprised an individualized management program, involving nurse-led education, and management plan with medical specialist oversight. The outcome, participants’ knowledge of secondary prevention medications at 12 months, was assessed using questionnaires. A score of ≥5 was considered as good knowledge. Effectiveness of the intervention on knowledge of medications was determined using logistic regression.

**Results:**

Between May 2014 and January 2015, 142 consecutive participants from the main trial were included in this sub-study, 64 to usual care and 78 to the intervention (median age 68.9 years, 68% males, and 79% ischemic stroke). In multivariable analyses, we found no significant difference between intervention groups in knowledge of medications. Factors independently associated with good knowledge (score ≥5) at 12 months included higher socioeconomic position (OR 4.79, 95% CI 1.76, 13.07), greater functional ability (OR 1.69, 95% CI 1.17, 2.45), being married/living with a partner (OR 3.12, 95% CI 1.10, 8.87), and using instructions on pill bottle/package as an administration aid (OR 4.82, 95% CI 1.76, 13.22). Being aged ≥65 years was associated with poorer knowledge of medications (OR 0.24, 95% CI 0.08, 0.71), while knowledge was worse among those taking three medications (OR 0.15, 95% CI 0.03, 0.66) or ≥4 medications (OR 0.09, 95% CI 0.02, 0.44), when compared to participants taking fewer (≤2) prevention medications.

**Conclusion:**

There was no evidence that the nurse-led intervention was effective for improving knowledge of secondary prevention medications in patients with stroke/TIA at 12 months. However, older patients and those taking more medications should be particularly targeted for more intensive education.

**Trial registration:**

Australian New Zealand Clinical Trials Registry (ACTRN12688000166370).

## Introduction

Use of evidence-based pharmacological therapies is a recognized strategy for controlling vascular risk factors in patients with stroke (1–3). In patients with chronic diseases, such as stroke, a major factor associated with adherence to medications is adequate knowledge regarding the often complex medication regimen (4). This includes knowing the name, administration, handling, and potential side effects of the medications (5). Moreover, authors have reported associations between knowledge of medications and control of blood glucose in patients with diabetes (6), control of blood pressure in those with hypertension (7), and reduction of adverse outcomes in patients with vascular disease (8). However, there is evidence that information needs of patients with chronic diseases, regarding their medications, are not being met (9).

To empower patients with chronic diseases, it is recommended that education and counseling on use of medications is initiated during their acute hospital stay (10). This is usually complemented by long-term interventions to facilitate effective use of medications post-discharge (11). Primary care providers, including nurses and pharmacists, have important roles in implementing these strategies.

Many studies have been conducted to determine the effectiveness of educational strategies to improve adherence to medications in the treatment of chronic diseases (12). However, limited data exist on effective interventions to improve knowledge of medications in this high-risk population. In patients with hypertension, there was no evidence that a nursing intervention improved knowledge of preventive medications after 12-month follow-up (13). The benefit of an intervention to improve knowledge of medications in people with stroke has been reported previously (14). However, this evidence is limited by a weak methodological approach. Clearly, a more robust approach would provide more reliable evidence on this topic, this being the rationale for our study. We investigated the effectiveness of a nurse-led individualized management program for improving knowledge of secondary prevention medications in patients with stroke or transient ischemic attack (TIA). We hypothesized that survivors of stroke or TIA who received our intervention would have better knowledge of secondary prevention therapies, when compared to those receiving usual care.

## Materials and Methods

### Trial Design and Participants

**Figure 1:**
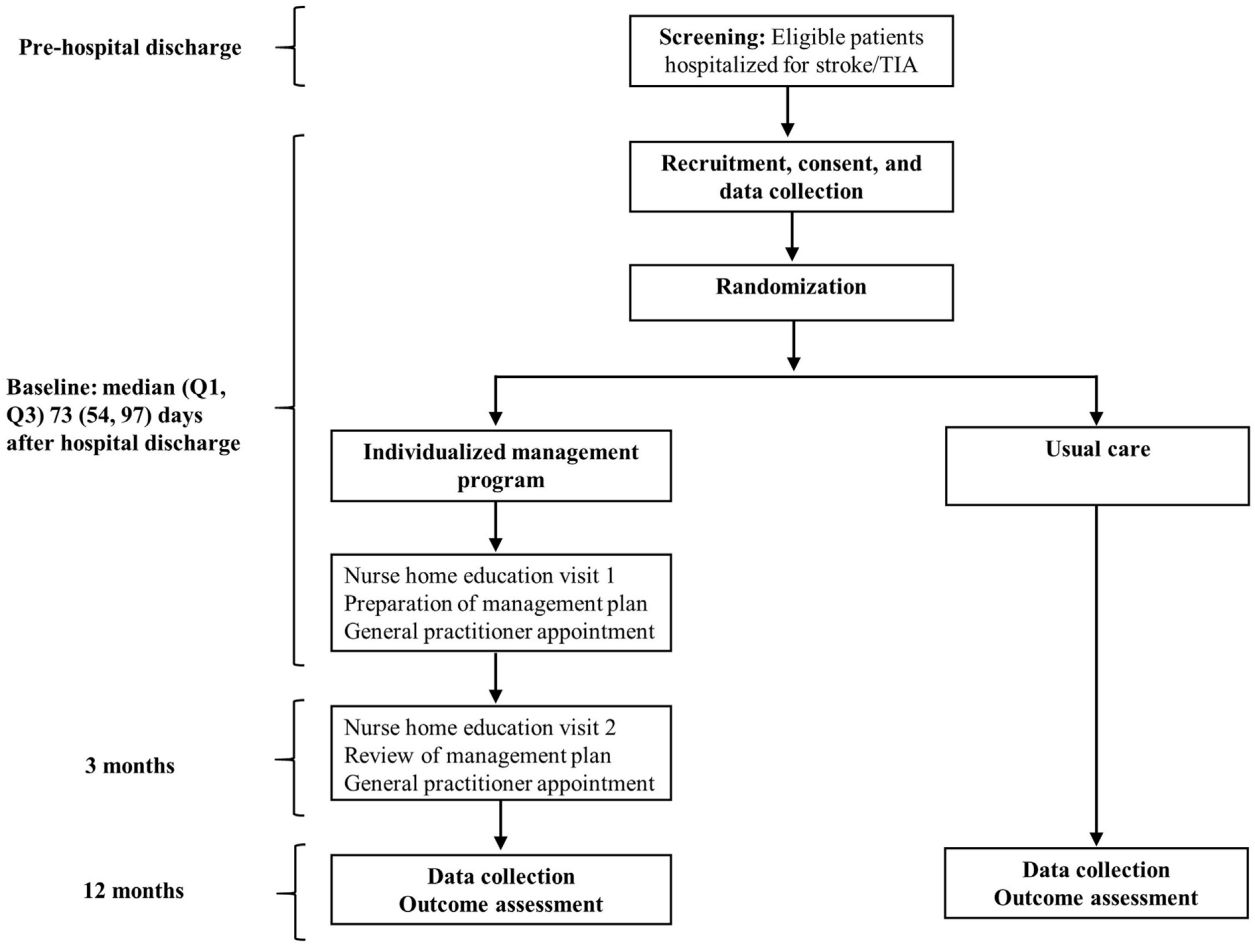
**Trial design**.

**Figure 2:**
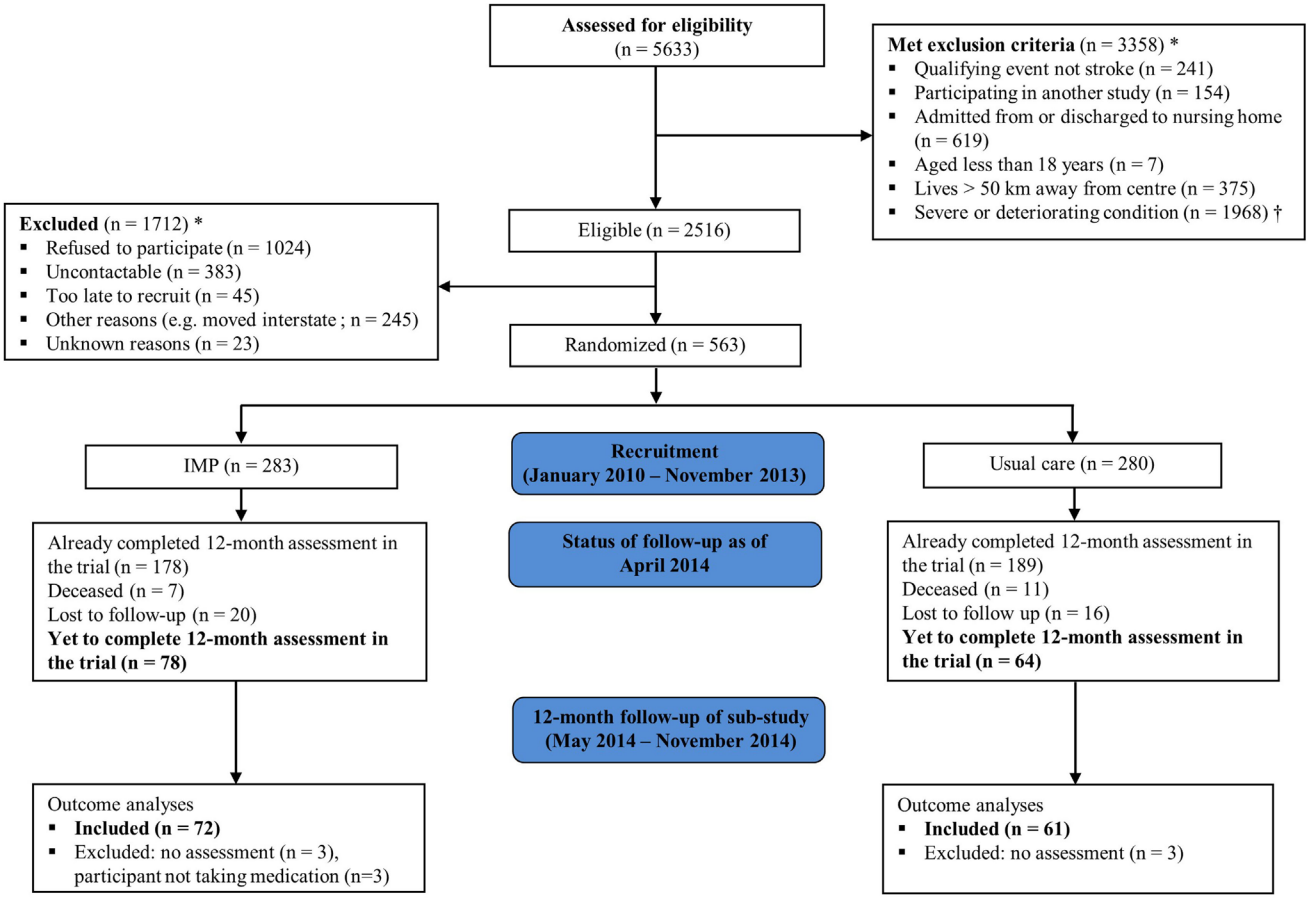
**Flow chart of participants through the study**. IMP, individualized management program; *Participants may have more than one reason for exclusion; ^†^Severe or deteriorating condition includes concurrent illness, dementia, palliated, or deceased.

This was a prospective sub-study of the Shared Team Approach between Nurses and Doctors for Improved Risk Factor Management (STAND FIRM), a multicenter cluster-randomized controlled trial, in patients with stroke/TIA. A detailed description of the design for the STAND FIRM trial has been published elsewhere (15). Briefly, participants were recruited from four teaching hospitals in Melbourne, Australia: Monash Medical Center, Alfred Hospital, Box Hill Hospital, and Dandenong Hospital, between January 2010 and November 2013. Eligible patients were adults (aged ≥18 years) hospitalized for stroke/TIA, and living within 50 km of the closest recruitment hospital (Figures 1 and 2). We excluded patients recruited to another trial, admitted from or discharged to a nursing home, or presenting with worsening health condition.

Patients who met the eligibility criteria were randomized to either receive usual care or an individualized management program in addition to usual care. A computer-generated, blocked randomization procedure was undertaken to ensure that each recruitment hospital had a balance of patients in each group. This was to remove any potential treatment bias as post-acute treatment may vary somewhat at each recruitment hospital. Further, as general practitioners (GPs) play a significant role in delivering the intervention, randomization was clustered by general practice to minimize contamination between the intervention and usual care groups. As a result, GPs nominated by the participants, and the practice in which they belonged, were randomly allocated to either provide usual care only, or the intervention in addition to usual care. This meant that a GP participating in the trial, or the practice to which the GP belonged, could only treat patients in one of the two treatment groups. As a result, GPs and practices nominated by newly recruited patients were checked against a list of all GPs and practices already participating in the study. If a GP or practice nominated by a newly recruited participant already had a patient in the study, then the participant was allocated to the treatment group in which the GP or practice belonged. However, if such GP or practice did not already have a patient in the study, then the GP or practice was randomly allocated to the next random allocation within the block.

In May 2014, a sub-study was initiated to objectively evaluate the effectiveness of the nurse education component of the intervention. This initiative was borne out of lack of robust or reliable evidence on effective interventions to improve knowledge of medications in people with chronic diseases. In the present sub-study, we enrolled consecutive participants who were yet to undertake 12-month assessments in the STAND FIRM trial. Participants were enrolled from both study arms and all four recruitment hospitals, and no new selection criteria were used. Ethics approval was obtained from all participating hospitals and Monash University (HREC number 2011000331), and written informed consent was obtained from all participants. STAND FIRM trial is registered with the Australian New Zealand Clinical Trials Registry (ACTRN12680000166370).

### Usual Care

Participants randomized to usual care received standard care available in the stroke prevention clinic of the participating hospitals and in general practice. Standard care may have involved provision of education and advice on secondary prevention by care providers, including community-based pharmacists.

### Intervention

The intervention group received an individualized management program, comprising a chronic disease management (CDM) plan and two home visits by nurses to provide tailored education for secondary prevention, in addition to usual care (Figure 1). An unblinded nurse, in consultation with a stroke specialist, specifically tailored the CDM plan to each participant using health information obtained by a blinded assessor at baseline assessment. The CDM plan comprised clear health goals for secondary prevention, including adherence to recommended therapies. This plan was then provided to the GP to facilitate the care of their patients.

Prior to involving GPs in the intervention, an unblinded nurse conducted an in-home visit with participants to discuss their health goals, provide tailored education, and discuss strategies to overcome barriers. Specifically, the nurse reviewed medications prescribed for secondary prevention and provided formal education or counseling on the use of these medications. Information provided on medications included dosage and time of administration, benefits of adherence, and self-management skills, such as identifying and managing potential side effects. Nurse education was facilitated by the use of brochures provided by Australia’s Stroke Foundation (16), and standard syllabus for education on secondary prevention of stroke (Data Sheet S1 in Supplementary Material). The syllabus comprised a component on prevention medications (Page 6; Data Sheet S1 in Supplementary Material). Participants were also invited to ask questions about their medications or general recovery. Finally, the nurse organized appointments for participants to discuss their CDM plan with their GP. At 3 months after baseline, the CDM plan was reviewed and the education visit was repeated, taking into account any changes in medication regimen.

### Baseline and Follow-up Assessments

Baseline data on details of the stroke were obtained from the hospital records of the patients, while demographic data, details of prescribed medications, and standardized assessment of mental and functional status were obtained by a blinded assessor during an in-home patient visit (median 10 weeks post-discharge). The London Handicap Scale (LHS) was used to measure functional disability, and the Hospital Anxiety and Depression Scale (HADS) for mood disorders, at baseline, 3, and 12 months.

### Outcome Assessment

The study outcome comprised participants’ knowledge of their medications for secondary prevention at 12 months, ascertained by blinded assessors. Recommended therapies assessed included three categories: antihypertensive, antithrombotic, and cholesterol-lowering medications. Knowledge was assessed using a modified version of the McPherson index (6, 17, 18). The items comprised name of the medication, purpose, mechanism of action, time of administration, knowledge of side effects, and what to do when side effects occurred or doses were missed. Participants were assessed on only the categories of medications they were taking at 12 months; in those taking more than one type of medication in each category, one was randomly selected for assessment.

For each of the seven items assessed, a correct answer was scored 1, and an incorrect answer 0 (Table 1). The overall score for each item was the sum of scores for an item divided by the number of categories of medications used, giving a maximum score of 1 for each item. A similar approach was used for overall knowledge of medications, but used all items and all categories (possible range 0–7). Based on data from previous studies (6, 17), and median knowledge score in our study population, an individual item score of 1 and a composite score of ≥5 were considered as good knowledge.

**Table 1:** **Scoring algorithm for knowledge of medications**.

Knowledge category	Medication category^a^			
	Antihypertensive	Antithrombotic	Cholesterol lowering	Score
Name	1	1	–	2/2 = 1
Purpose	1	0	–	1/2 = 0.5
Mechanism of action	1	1	–	2/2 = 1
Time of Administration	1	0	–	1/2 = 0.5
Knowledge of side effects	1	1	–	2/2 = 1
What to do when there are side effects	1	0	–	1/2 = 0.5
What to do when doses are missed	1	1	–	2/2 = 1
Total score	7	4	–	11/2 = 5.5

*^a^Possible score for each item is 0 for no knowledge and 1 for knowledge*.

*The above example is for a person who takes an antihypertensive and an antithrombotic agent, but does not take a cholesterol-lowering medication*.

### Statistical Analysis

Because of limited data on knowledge of medications both in survivors of stroke and the general population, a reliable estimate of sample size was not possible. However, in this study, outcome data were obtained from as many participants as possible in the STAND FIRM cohort.

Continuous variables were summarized as medians and quartiles (Q1, Q3), while categorical variables were summarized as frequency counts and percentages. In order to assess balance between the intervention and control groups at baseline, participant characteristics were compared using Wilcoxon rank sum test for continuous variables, and χ^2^ test for categorical variables. In cases where cell frequencies were <5, Fisher’s exact test was used to compare categorical variables.

All outcome analyses were performed based on intention-to-treat. Regression analyses were used to determine the effect of the intervention on knowledge of medications. A univariable logistic regression model was used when knowledge was defined as a categorical variable, i.e., a composite score of ≥5 for good knowledge and <5 for poor knowledge. A univariable linear regression model was used when knowledge was defined as a continuous variable. In the adjusted analyses, multivariable regression models were conducted using stepwise selection procedure. The full multivariable models included baseline variables such as age, sex, marital status, socioeconomic position, educational attainment, type of stroke, and medical history. The models also included variables obtained at 12 months, such as mental and functional status, number of prescribed secondary prevention medications, length of use of prevention medications, method by which medications are packaged or provided by pharmacist, and method of keeping track of medications. Apart from age and sex, that were fixed, only variables with a *P*-value of <0.05 were retained in the final model. Similar procedures were used to determine the effect of the intervention on the six measured attributes of knowledge of medications.

To identify patient factors independently associated with knowledge of medications, a stepwise logistic regression model was constructed using methods similar to those stated above. To further explain the results of the outcome analyses, a sensitivity analysis was conducted using a per-protocol dataset. The per-protocol dataset comprised data from only participants with no deviation from the protocol, i.e., participants who received nurse home education visits both at baseline and 3 months. Analyses were not adjusted for any effect of clustering by general practice given the few number of participants in each cluster (average two participants per general practice). All analyses were conducted using STATA IC (12.0). A two-sided *P*-value of <0.05 was considered statistically significant.

## Results

### Participant Flow and Baseline Characteristics

A total of 563 participants were recruited into the STAND FIRM trial, of whom 142 (25%) consecutive participants were eligible (i.e., alive and had not undertaken 12-month assessment at the time of commencement of the present sub-study), and were subsequently enrolled. These comprised 78 participants in the intervention group and 64 in the control group, median age 68.9 years, 68% male, and 79% ischemic stroke (Figure 2). Participants who were not enrolled were more often depressed than those who were enrolled (15 vs. 9%, *P* = 0.044; Table 2). No other difference in baseline characteristics was detected between participants who were enrolled and those not enrolled in this sub-study. The intervention group had greater socioeconomic position than the control group (58 vs. 39%, *P* = 0.007). There was no other difference in baseline characteristics between study groups.

**Table 2:** **Baseline characteristics**.

Characteristics	STAND FIRM cohort (*n* = 533)			
	Not enrolled in sub-study (*n* = 391)	Enrolled in sub-study (*n* = 142)		
		Total	Intervention (*n* = 78)	Control (*n* = 64)
Aged ≥65 years	246 (62.9)	88 (62.0)	47 (60.3)	41 (64.1)
Female	139 (35.6)	45 (31.7)	20 (25.6)	25 (39.1)
Vocational or higher education	195 (49.9)	84 (59.2)	48 (61.5)	36 (56.3)
High socioeconomic position^a^	204 (52.2)	64 (45.1)	45 (57.7)	25 (39.1)
Married or living with partner	256 (65.5)	100 (70.4)	52 (66.7)	48 (75.0)
**Type of stroke**				
Ischemic stroke	303 (77.5)	112 (78.9)	60 (76.9)	52 (81.3)
Intracerebral hemorrhage	31 (7.9)	11 (7.8)	7 (9.0)	4 (6.3)
Transient ischemic attack	57 (14.9)	19 (13.4)	11 (14.1)	8 (12.5)
Recurrent stroke	59 (15.1)	18 (12.7)	9 (11.5)	9 (14.1)
≥2 comorbidities	214 (54.7)	71 (50.0)	34 (43.6)	37 (57.8)
**Prescribed secondary prevention medications**				
Total [median (Q1, Q3)]	2 (2, 3)	2 (2, 3)	3 (3, 4)	3 (3, 4)
≤2	86 (22.0)	35 (24.7)	19 (24.4)	16 (25.0)
3	128 (32.7)	55 (38.7)	33 (42.3)	22 (34.4)
≥4	177 (45.3)	52 (36.6)	26 (33.3)	26 (40.6)
Antihypertensive	330 (84.4)	115 (81.0)	64 (82.1)	51 (79.7)
Cholesterol-lowering	338 (86.5)	124 (87.3)	69 (88.5)	55 (85.9)
Antithrombotic	355 (90.8)	130 (91.6)	70 (89.7)	60 (93.8)
Disability [median LHS score (Q1, Q3)]	0.85 (0.73, 0.93)	0.86 (0.80, 0.97)	0.86 (0.80, 0.97)	0.86 (0.75, 0.97)
Depressed (HADS >7)^b^	58 (14.9)	12 (8.5)	6 (7.7)	6 (9.4)
Anxious (HADS >7)	77 (19.8)	25 (17.6)	14 (18.0)	11 (17.2)

*^a^Statistical difference in socioeconomic position between intervention and control groups (*P* = 0.007)*.

*^b^Statistical difference in proportion of participants with depression between participants enrolled and not enrolled (*P* = 0.044)*.

*Data are expressed as frequency and proportion unless otherwise stated*.

*LHS, London Handicap Scale; HADS, Hospital Anxiety and Depression Scale*.

After the commencement of the present sub-study, there were no losses to follow-up. However, 6 (4%) of the 142 consecutive participants enrolled to this sub-study were not assessed for outcome measures as a result of logistical issues, while another 3 (2%) were not taking prevention medications at the 12-month follow-up. These participants were excluded from outcome analyses as there were no data on knowledge of medications. Therefore, 133 (94%) participants were assessed at 12 months, and were included in the outcome analyses. A relative provided information for one participant, but in all other instances, the information was obtained directly from participants.

### Outcome Analyses

Overall, at 12 months, the median (Q1, Q3) score for knowledge of medications was 5.3 (3.7, 6.7), and 54% of participants had good knowledge (score ≥5). In multivariable logistic regression (knowledge treated as categorical variable) and linear regression (knowledge treated as continuous variable) analyses, there was no detectable difference in knowledge of medications for secondary prevention between intervention and control groups at 12 months (Table 3). Similar results were obtained for each of the seven items that comprised the composite score for medication knowledge (Table 3). In the per-protocol analyses, comprising 67 participants in the intervention group and 61 participants in the control group, there was no detectable difference between treatment groups in both the overall knowledge of medications, and the measured attributes of knowledge of medications (Table 4).

**Table 3:** **Univariable and multivariable analyses of the effect of intervention on knowledge of medications**.

Medication knowledge	Participants obtaining optimal score^a^		Univariable	Multivariable^d^	
	Intervention *N* (%) (*n* = 72)	Control *N* (%) (*n* = 61)	(OR, 95% CI)	(OR, 95% CI)	*P*-value
**Individual item score (optimal score = 1)^b^**					
Name of medication	48 (66.7)	38 (62.3)	1.21 (0.59, 2.47)	1.03 (0.46, 2.34)	0.938
Reasons for administration	42 (58.3)	35 (57.4)	1.04 (0.52, 2.07)	0.87 (0.39, 1.97)	0.751
Mechanism of administration	40 (55.6)	33 (54.1)	1.06 (0.53, 2.10)	1.17 (0.49, 2.78)	0.730
Timing of medication	64 (88.9)	57 (93.4)	0.56 (0.16, 1.96)	0.44 (0.10, 1.20)	0.219
Side effect	7 (9.7)	5 (8.2)	1.20 (0.36, 4.01)	1.27 (0.32, 4.98)	0.736
**What to do when**					
Medication side effects occur	60 (83.3)	47 (77.1)	1.48 (0.63, 3.52)	1.58 (0.62, 4.00)	0.336
Dose of medication is missed	56 (77.8)	53 (85.3)	0.61 (0.24, 1.49)	0.71 (0.26, 1.95)	0.511
**Composite score^c^**					
Median (Q1, Q3)	5.6 (3.6, 6.0)	5.0 (3.7, 6.7)	0.92 (0.63, 1.85)	0.89 (0.56, 1.41)^e^	0.612
Optimal (≥5)	47 (65.3)	37 (60.7)	1.22 (0.60, 2.47)	1.10 (0.42, 2.92)	0.841

*^a^Analyses were restricted to 133 patients who were taking medications at 12 months*.

*^b^Item score was calculated as the sum of scores for an item divided by the number of medications assessed*.

*^c^Composite score was calculated as the sum of scores for all items divided by the number of categories of medications used*.

*^d^Adjusted for type of stroke, medical history, demographic, socioeconomic, mental and functional status, number of prescribed prevention medications, length of use of prevention medications, method by which medications are packaged/provided by pharmacist, and self-reported method(s) of keeping track of medications*.

*^e^Exponentiated coefficient obtained from the stepwise multivariable linear regression analyses*.

*OR, odds ratio; CI, confidence interval*.

**Table 4:** **Per protocol analyses of the effect of intervention on knowledge of medications**.

Medication knowledge	Participants obtaining optimal score^a^		Univariable	Multivariable^d^	
	Intervention *N* (%) (*n* = 67)	Control *N* (%) (*n* = 61)	(OR, 95% CI)	(OR, 95% CI)	*P-*value
**Individual item score (optimal score = 1)^b^**					
Name of medication	43 (64.2)	38 (62.3)	1.08 (0.53, 2.23)	1.02 (0.43, 2.39)	0.967
Reasons for administration	38 (56.7)	35 (57.4)	0.97 (0.48, 1.96)	0.83 (0.35, 1.93)	0.659
Mechanism of administration	36 (53.7)	33 (54.1)	0.99 (0.49, 1.98)	1.00 (0.41, 2.45)	0.996
Timing of medication	60 (89.6)	57 (93.4)	0.60 (0.17, 2.17)	0.51 (0.14, 1.91)	0.321
Side effect	7 (10.5)	5 (8.2)	1.31 (0.39, 4.36)	1.47 (0.38, 5.77)	0.579
**What to do when**					
Medication side effects occur	55 (82.1)	47 (77.1)	1.37 (0.58, 3.24)	1.47 (0.57, 3.79)	0.420
Dose of medication is missed	52 (77.6)	52 (85.3)	0.60 (0.24, 1.49)	0.74 (0.27, 2.06)	0.568
**Composite score^c^**					
Median (Q1, Q3)	5.5 (3.5, 6.0)	5.0 (3.7, 6.7)	1.02 (0.59, 1.78)	1.01 (0.38, 2.71)^e^	0.980
Optimal (≥5)	43 (64.2)	37 (60.7)	1.16 (0.57, 2.38)	0.87 (0.54, 1.40)	0.566

*^a^Analyses were restricted to 128 patients who were taking medications at 12 months*.

*^b^Item score was calculated as the sum of scores for an item divided by the number of medications assessed*.

*^c^Composite score was calculated as the sum of scores for all items divided by the number of categories of medications used*.

*^d^Adjusted for type of stroke, medical history, demographic, socioeconomic, mental and functional status, number of prescribed prevention medications, length of use of prevention medications, method by which medications are packaged/provided by pharmacist, and self-reported method(s) of keeping track of medications*.

*^e^Exponentiated coefficient obtained from the stepwise multivariable linear regression analyses*.

*OR, odds ratio; CI, confidence interval*.

Factors independently associated with good knowledge of medications (score ≥5) at 12 months (Table 5) were having a higher socioeconomic position (OR 4.79, 95% CI 1.76, 13.07), greater functional ability (OR 1.69, 95% CI 1.17, 2.45), being married/living with a partner (OR 3.12, 95% CI 1.10, 8.87), or using instructions on pill bottle/package as an administration aid (OR 4.82, 95% CI 1.76, 13.22). In contrast, being aged ≥65 years was associated with poorer knowledge of medications (OR 0.24, 95% CI 0.08, 0.71). Moreover, taking a greater number of prevention medications was also associated with poorer knowledge of these medications, i.e., when compared to participants taking fewer (≤2) prevention medications, knowledge was worse among those taking three medications (OR 0.15, 95% CI 0.03, 0.66) or four or more medications (OR 0.09, 95% CI 0.02, 0.44).

**Table 5:** **Factors associated with knowledge of medications**.

Characteristics	Univariable		Multivariable^a^	
	OR (95% CI)	*P-*value	OR (95% CI)	*P*-value
Intervention	1.22 (0.60, 2.47)	0.582	1.10 (0.42, 2.92)	0.841
Aged ≥65 years	0.27 (0.12, 0.61)	0.002	0.24 (0.08, 0.71)	0.010
Male	0.83 (0.39, 1.77)	0.621	2.69 (0.83, 8.76)	0.100
Vocational or higher degree	1.88 (0.91, 3.84)	0.085	–	–
High socioeconomic position^b^	2.64 (1.27, 5.47)	0.009	4.79 (1.76, 13.07)	0.002
Married or living with partner	3.76 (1.72, 8.21)	0.001	3.12 (1.10, 8.87)	0.033
Recurrent stroke	0.62 (0.22, 1.72)	0.353	–	–
≥2 comorbidities	0.68 (0.34, 1.39)	0.290	–	–
**Prescribed secondary prevention medications**				
≤2	1.0		1.0	–
3	0.21 (0.06, 0.70)	0.011	0.15 (0.03, 0.66)	0.013
≥4	0.17 (0.05, 0.55)	0.003	0.09 (0.02, 0.44)	0.003
Provided medications in pill bottle/package	11.87 (2.51, 56.20)	0.002	–	–
Keep track of medications using instructions on pill bottle/package	4.20 (1.96, 8.98)	<0.001	4.82 (1.76, 13.22)	0.002
Using prevention medications for ≥2 years	0.99 (0.49, 2.01)	0.972	–	–
Increased ability (per 0.1 LHS)	1.68 (1.29, 2.21)	<0.001	1.69 (1.17, 2.45)	0.006
Depressed (HADS >7)	0.47 (0.17, 1.31)	0.147	–	–
Anxious (HADS >7)	1.52 (0.58, 3.98)	0.391	–	–

*^a^Multivariable model was adjusted for all other factors in the column*.

*^b^Socioeconomic position determined using Australian Socioeconomic Indexes for Areas based on postcode*.

*LHS, London Handicap Scale; HADS, Hospital Anxiety and Depression Scale; OR, odds ratio; CI, confidence interval*.

## Discussion

The educational intervention that we investigated was an integral part of a comprehensive, multifaceted intervention for secondary prevention of stroke. We adopted structured and tailored education of participants to improve knowledge of medications and skills for medication management. Despite this, we found no evidence for better knowledge of secondary prevention medications in the intervention group than control. Similarly, our intervention did not improve any of the measured attributes of medication knowledge relative to current practice.

Our finding is in contrast with that of a similar multifaceted study conducted in Israel (14). In that study, a tailored nursing intervention improved knowledge of important attributes of medication knowledge such as dosage, side effects, and what to do in response to side effects, at 3 months and 6 months post-stroke. Moreover, knowledge of the timing of medications was significantly better in the intervention group than controls at 3 months after stroke. However, these findings may have been potentially biased by the non-randomized nature of the study design. Importantly, this difference in methodological approach hinders reliable comparison between the findings reported in the Israeli study and those observed in our study. Therefore, further research is needed in order to confirm the results of the present study, and to appropriately inform practice.

The lack of effect of the intervention undertaken in our study highlights the challenges in meeting medication information needs of survivors of stroke, and emphasizes a clear need to optimize education strategies on use of secondary prevention medications. This could involve education tailored to individual learning abilities and skills. For instance, it is known that survivors of stroke are often less receptive to knowledge of secondary prevention measures than people with other conditions, as a result of their neurological deficits (19), or cognitive deficits related to old age (9). Therefore, specific education strategies or methods to overcome these learning abilities should be adopted.

Another possible explanation for the lack of effect of our intervention may be the long delay between education sessions (occurring at baseline and 3 months) and the outcome assessments (occurring at 12 months). The impact of the intervention may have diminished over this period, as any education or knowledge acquired may be forgotten over time. Strategies to reduce this loss of knowledge could involve the use of reminders or more frequent delivery of education or follow-up, to reinforce information on use of medications, so that knowledge is maintained in the long term. Pharmacists can play a crucial role as reminders of important prescription information when providing refills after hospital discharge, although the impact of such intervention on patient medication knowledge is unknown.

In the present study, although ≥80% of participants knew what to do when experiencing medication side effects, they rarely knew what side effects could occur (<10%). Similarly, in Israel, more than 70% of participants knew what to do when experiencing side effects, but knowledge of side effects was only moderate (14). Knowledge of side effects is very important in secondary prevention, facilitating prompt response to medication-related adverse reactions (20), or reducing fear of severe medication-related complications (21).

Our observation that 35% of our participants had incorrect or no knowledge of fundamental attributes of their medications, such as the names of the medications, was better than that reported in an Israeli study (14). However, better knowledge of reasons for administration of medications was reported in other patient groups (≥82%) (22), than those found in our study (58%). It is important to acknowledge that our estimates may not be easily compared with those reported in previous studies because of differences in the methods used to assess medication knowledge. However, these findings still highlight the gaps in meeting information needs regarding medication regimens in patients with stroke.

Similar to our findings, others have reported that older age having a low income, being disabled, and increasing polypharmacy, are associated with poor knowledge of medications in other patient groups (4, 17). These patients are at greater risk of poor knowledge and are more likely to benefit from targeted and intensive intervention to improve knowledge of medications. Therefore, these factors should be taken into consideration when designing education strategies for secondary prevention of stroke.

An important and novel finding of this study is the importance of instructions on the prescription container as a major source of information on medications. Indeed, in the present study, participants that reported using instructions on pill bottles/package to keep track of their medications had significantly better knowledge of their medications than those using other dose administration aids, such as dosette boxes and bubble packs. To further explain this finding, we investigated the possibility of type of dose administration aid being a surrogate for the number of comorbidities presented by an individual. However, when number of comorbidities was forced into the regression model, there was no substantial change in the original estimates (odds ratios) observed for the variables retained in the model. Therefore, the role of methods by which medications are provided for improving general long-term medication management remains unclear.

An important limitation of our study is the lack of baseline data on knowledge of medications to determine a change over time between groups. This limitation arose because this sub-study was conceived after the commencement of the main trial to fill an important evidence gap on the improvement of medication knowledge in secondary prevention of stroke. However, the level of balance observed between study groups at baseline is reflective of the successful randomization procedure used in this study. Therefore, it is unlikely there was any significant difference in knowledge between study groups at baseline. Our study is also limited by a small sample size that could affect the robustness of the estimates on the efficacy of our intervention, and limit the power to identify factors associated with better knowledge of medications. Our findings may not be representative of the wider stroke population as a result of potential selection bias. For instance, the present study was conducted in community-dwelling survivors of stroke or TIA, thereby excluding people with severe stroke. Moreover, only 22% of the patients who met eligibility criteria were enrolled to the main trial. However, it is important to acknowledge that poor rate of recruitment is a common phenomenon in stroke trials (23). A major strength of our study is the robust study design. When compared to similar studies (14, 22), the randomized controlled design used in the present study limited potential bias from confounding factors. Therefore, our findings provide a more reliable basis for further research.

In conclusion, our study did not provide evidence for the effectiveness of a nurse-led, community-based educational intervention for improving knowledge of secondary prevention medications in survivors of stroke or TIA. As limited data exist on this topic, our findings provide a rationale for further investigating strategies to improve knowledge of medication in this high-risk population. Importantly, we have identified sub-groups of patients who are at greater risk of poor knowledge of their secondary prevention medications, and are therefore groups most likely to benefit from more intensive educational intervention.

## Author Contributions

AT is the principal investigator. AT, DC, VS, MO, MN, CB, RG, SF, TP, and JF designed the study. MO and JK coordinated the collection and management of data. MO undertook the statistical analyses and wrote the first draft of the report. All the authors had full access to all of the data, contributed to the interpretation of results, provided intellectual input to the manuscript, and approved the final version of the manuscript. All the authors are accountable for all aspects of this work, including the accuracy and integrity of the data reported in this work.

## Conflict of Interest Statement

TP has received honoraria for presentations given for Bayer, Boehringer Ingelheim, Genzyme, Pfizer, and Bristol-Myers Squibb. None of the other authors have relationships that might have an interest in this work.

## References

[1] Lakhan S Sapko M. Blood pressure lowering treatment for preventing stroke recurrence: a systematic review and meta-analysis. Int Arch Med (2009) 2:30. 10.1186/1755-7682-2-30 19843330 PMC2771000

[2] Baigent C Sudlow C Collins R Peto R Antithrombotic Trialists’ Collaboration. Collaborative meta-analysis of randomised trials of antiplatelet therapy for prevention of death, myocardial infarction, and stroke in high risk patients. BMJ (2002) 324:71–86. 10.1136/bmj.324.7329.71 11786451 PMC64503

[3] Amarenco P Labreuche J. Lipid management in the prevention of stroke: review and updated meta-analysis of statins for stroke prevention. Lancet Neurol (2009) 8:453–63. 10.1016/S1474-4422(09)70058-4 19375663

[4] Burge S White D Bajorek E Bazaldua O Trevino J Albright T Correlates of medication knowledge and adherence: findings from the residency research network of South Texas. Fam Med (2005) 37:712–8. 16273450

[5] Borgsteede SD Karapinar-Carkit F Hoffmann E Zoer J van den Bemt PM. Information needs about medication according to patients discharged from a general hospital. Patient Educ Couns (2011) 83:22–8. 10.1016/j.pec.2010.05.020 20554422

[6] McPherson ML Smith SW Powers A Zuckerman IH. Association between diabetes patients’ knowledge about medications and their blood glucose control. Res Social Adm Pharm (2008) 4:37–45. 10.1016/j.sapharm.2007.01.002 18342821

[7] Abu Farha R Basheti I Abu Al Ruz H Alsaleh A AbuRuz S Assessment of drug-related problems and their impact on blood pressure control in patients with hypertension. Eur J Hosp Pharm (2016) 23:126–30. 10.1136/ejhpharm-2015-000712 PMC6451509 31156832

[8] Hope CJ Wu J Tu W Young J Murray MD. Association of medication adherence, knowledge, and skills with emergency department visits by adults 50 years or older with congestive heart failure. Am J Health Syst Pharm (2004) 61:2043–9. 15509127 10.1093/ajhp/61.19.2043

[9] Marek KD Antle L Medication management of the community-dwelling older adult. In: Hughes RG, editor. Patient Safety and Quality: An Evidence-Based Handbook for Nurses. Advances in Patient Safety. Rockville, MD: US Agency for Healthcare Research and Quality (2008) 1 p. 499–536.

[10] Rahiman A Saver JL Porter V Buxton W McNair N Razinia T In-hospital initiation of secondary prevention is associated with improved vascular outcomes at 3 months. J Stroke Cerebrovasc Dis (2008) 17:5–8. 10.1016/j.jstrokecerebrovasdis.2007.09.004 18190814

[11] Cameron JI Gignac MA “Timing it right”: a conceptual framework for addressing the support needs of family caregivers to stroke survivors from the hospital to the home. Patient Educ Couns (2008) 70:305–14. 10.1016/j.pec.2007.10.020 18155388

[12] Kripalani S Yao XM Haynes RB Interventions to enhance medication adherence in chronic medical conditions – a systematic review. Arch Intern Med (2007) 167:540–50. 10.1001/archinte.167.6.540 17389285

[13] Guirado EA Ribera EP Huergo VP Borras JM ADIEHTA Group. Knowledge and adherence to antihypertensive therapy in primary care: results of a randomized trial. Gac Sanit (2011) 25:62–7. 10.1016/j.gaceta.2010.09.015 21354671

[14] Nir Z Weisel-Eichler A. Improving knowledge and skills for use of medication by patients after stroke: evaluation of a nursing intervention. Am J Phys Med Rehabil (2006) 85:582–92. 10.1097/01.phm.0000223227.51120.4d 16788389

[15] Thrift AG Srikanth VK Nelson MR Kim J Fitzgerald SM Gerraty RP Risk factor management in survivors of stroke: a double-blind, cluster-randomized, controlled trial. Int J Stroke (2014) 9:652–7. 10.1111/j.1747-4949.2012.00933.x 23231528

[16] Stroke Foundation. Medication after Stroke Fact Sheet. Melbourne: Stroke Foundation (2016). Available from: https://strokefoundation.com.au/About-Stroke/Help-after-stroke/Stroke-resources-and-fact-sheets/Medication-after-stroke-fact-sheet

[17] Okuyan B Sancar M Izzettin FV. Assessment of medication knowledge and adherence among patients under oral chronic medication treatment in community pharmacy settings. Pharmacoepidemiol Drug Saf (2013) 22:209–14. 10.1002/pds.3275 22514147

[18] Awwad O Akour A Al-Muhaissen S Morisky D. The influence of patients’ knowledge on adherence to their chronic medications: a cross-sectional study in Jordan. Int J Clin Pharm (2015) 37:504–10. 10.1007/s11096-015-0086-3 25708124

[19] Griffin LJ Hickey JV Considerations and strategies for educating stroke patients with neurological deficits. J Nurs Educ Pract (2013) 3:125–37. 10.5430/jnep.v3n8p125

[20] Gallacher K Morrison D Jani B MacDonald S May CR Montori VM Uncovering treatment burden as a key concept for stroke care: a systematic review of qualitative research. PLoS Med (2013) 10(6):e1001473. 10.1371/journal.pmed.1001473 23824703 PMC3692487

[21] Kim J Thrift AG Nelson MR Bladin CF Cadilhac DA. Personalized medicine and stroke prevention: where are we? Vasc Health Risk Manag (2015) 11:601–11. 10.2147/VHRM.S77571 26664130 PMC4671759

[22] Nurit P Bella BC Gila E Revital Z. Evaluation of a nursing intervention project to promote patient medication education. J Clin Nurs (2009) 18:2530–6. 10.1111/j.1365-2702.2009.02844.x 19694880

[23] Blanton S Morris DM Prettyman MG McCulloch K Redmond S Light KE Lessons learned in participant recruitment and retention: the EXCITE trial. Phys Ther (2006) 86:1520–33. 10.2522/ptj.20060091 17079752

