# Supplementary material for: Nurse-Led Intervention to Improve Knowledge of Medications in Survivors of Stroke or Transient Ischemic Attack: A Cluster Randomized Controlled Trial
Source: Front Neurol. 2016 Nov 18;7:205. doi: 10.3389/fneur.2016.00205 (PMC5114293; doi:10.3389/fneur.2016.00205)
Supplement: Supplementary file 1 [file Data_Sheet_1.DOCX]

Supplementary Material

**Nurse-led intervention to improve knowledge of medications in survivors of stroke or transient ischemic attack: a cluster randomized controlled trial**

**Muideen T Olaiya^1^, Dominique A Cadilhac^1,2^ Joosup Kim^1,2^, David Ung^1^, Mark R Nelson^3,4^, Velandai K Srikanth^1,3^, Christopher F Bladin^5^, Richard P Gerraty^6^, Sharyn M Fitzgerald^4^, Thanh Phan^1^, Judith Frayne^7^, Amanda G Thrift^1*^**

* Correspondence:

Professor Amanda G Thrift (amanda.thrift@monash.edu)

**Supplementary data. Template of STAND FIRM syllabus for nurse education visit**

**Summary for Patient Education Visit ID No:**

| **PATIENT DETAILS**  **Date of Birth:** **Gender:**  **Language Spoken:**  **Interpreter may be required:** | **GP DETAILS** |
| --- | --- |
| **Details of stroke**  Date of stroke:  Hospital Name: | Type of stroke: Ischaemic Stroke  Baseline Visit: |

| **MEDICAL HISTORY**  Diabetes  Hypertension  High cholesterol  Ischemic heart disease  Heart failure  Atrial fibrillation  **Other notes or comments relevant to the patient’s management plan:**  2001 – Heart Attack, Stent Inserted  2000 – Cataracts Both Eyes  2007 - Enlarged prostrate |
| --- |

| **FAMILY HISTORY (Reported by patient)**  Family history of heart disease  Family history of diabetes |
| --- |

| **MEDICATIONS**  Atorvastatin 40mg Daily  Karvezide 300/12.5mg Daily  Physiotens 0.2mg Twice Daily  Trajenta 5mg Daily  Norvasc 10mg Daily  Amaryl 4mg Daily  Diabex 1000mg Twice Daily  Betaloc 50mg Twice Daily  Jezil 600mg Twice Daily  Apidra 20 Units Daily  **ALLERGIES**  Nil Known |
| --- |

1. **What are your main concerns about having a stroke?**

If they are worried about having another stroke, discuss available options for support (use help after stroke card):

- National Stroke Foundation Helpline
- Encourage to discuss with GP
- If you experience symptoms dial 000 (and offer emergency card)

**Please document main issues for the participant and advice offered**

*Participant has heard of National Stroke Foundation from hospital. Discussed and showed participant the NSF fact sheets.*

*Participant has been with GP for a long time. Participant states GP is easy to discuss problems with. GP refers participant to specialists: endocrinologist, dermatologist, urologist, ophthalmologist, and neurologist.*

*Participant would call 000 if needed.*

If they are worried about living with the effects of stroke provide details about:

- Peer support (i.e. Stroke Association of Victoria, and Carers Australia)
- National Stroke Foundation Helpline
- Encourage to discuss with GP
- Beyond Blue Helpline
- Functional and impairment concerns (e.g. continence, speech, inability to walk, mood)

**Please document main issues for the participant and advice offered**

- Mobility
- Driving
- Speech/Communication
- Medication Use
- Support system at home
- Sexual Dysfunction

*1. Participant had 95% recovery post-stroke.*

*2. Participant was unable to drive initially post-stroke, but no issues now.*

*3. No issues with communication.*

*4. Participant is compliant with medication use, generally no issues. Participant is more aware of Insulin.*

*5. Participant is independent.*

*6. Participant has no issues.*

1. **Stroke Prevention Education**

**SMOKING**

Past smoker, ceased XX/XX/XX Cotinine: None detected

**Education**: The risk of heart disease halves within 12 months of quitting. The risk of stroke becomes the same as that for a never smoker after 5 years. Lung function improves within 3 months. Passive smoking is associated with a high risk of stroke to a similar level as for people who smoke, and exposure must be avoided in the home and other social environments.

**Goal**: Cease smoking (or remain smoke-free) and to steer clear of exposure to smoking (including in the home). Ban or restrict smoking by others in the home.

**Interest in quitting** (score out of 7; 7 = very keen): out of 7. What would need to happen to make you more keen to quit - say to give a score of 6 or 7 out of 7 instead?

**Confidence in quitting** (score out of 7; 7 = very confident): out of 7. What would be the hardest thing about quitting? What made it difficult to quit the last time you tried? What would need to happen to increase your confidence to 6 or 7? Explore and tackle barriers (e.g. withdrawal, stress reduction, weight control). Identify support, e.g. partner, GP. Refer to Quitline.

**Exposure to passive smoking.** Determine whether passive smoking is an issue in the home, car and elsewhere. Discuss strategies to avoid exposure to environmental tobacco smoke (e.g. banning smoking inside the home or in cars).

**Please document main issues for the participant and advice offered**

*Participant is not exposed to passive smoking.*

**BLOOD PRESSURE**

Measured blood pressure at home on XX/XX/XX; BP = 158/69 mmHg

Weight Assessed at home on XX/XX/XX; BMI =35.1 kg/m2 Waist (<80 F, < 94 M) = 126 cm Waist-Hip Ratio: 1.0

Participant has gained more than 10 kg since late teens or early twenties. Participant has tried to lose weight 1 time since late teens or early twenties.

Participant exercises vigorously (≥ 20 minutes) 0 times/wk. Walks 30 minutes or more 1 time/wk. Undertakes moderate-intensity activity that increases heart rate (≥30 minutes) 0 times/wk.

Participant adds salt to food at the table.

Salt excretion per day (Recommended range 40-220): 141 mmol/day

**Education**: It is recommended that all people who have had a stroke, regardless of blood pressure level, should receive blood pressure lowering therapy (unless you are prone to dizziness from very low blood pressure). Other ways to help reduce blood pressure include weight loss, increasing physical activity (to help with weight loss, improve cardiovascular fitness and mood, and reduce stress), reduce salt intake to less than 1 teaspoon per day, avoid processed food because these have high sources of salt and fat (particularly bread and ice cream), and increasing fruit and vegetable intake. GPs and pharmacists can provide support in these areas.

**Goal**: Lower blood pressure irrespective of baseline level. This minimizes damage to small blood vessels throughout the body.

**Snoring and or sleep apnoea:** Determine whether patient has ever been told that they have sleep apnoea. Are they being treated for sleep apnoea? Presence of sleep apnoea may increase blood pressure and treatment has been shown to lower blood pressure (Continuous Positive Airway Pressure (CPAP)).

**Please document main issues for the participant and advice offered**

*Participant sees GP once a month. GP takes BP measurement at each visit. Participant has been on BP medications for over 10 years. Participant is aware to also watch out for low BP.*

*Participant has gained weight since stroke, and knows connection of increasing weight with increasing BP, but has lacked incentive to reduce weight.*

*Participant is now looking at dietary changes and increasing exercise.*

*Participant does not think he has a snoring issue.*

**ATRIAL FIBRILLATION**

**Heart Rhythm**: Regular

**Education:** Irregular heart beat is associated with a very high risk of stroke and stroke recurrence. Medication can reduce this risk. Smoking may exacerbate this condition. Atrial fibrillation can be difficult to control. It is important to have your response to medication regularly monitored. Regular contact with your general practitioner may be needed. Teach the participant how to take their pulse and check for AF.

**Goal:** If AF present or suspected, seek medical advice (GP).

**Please document main issues for the participant and advice offered**

*Participant has had AF since 2000, sees cardiologist.*

*Participant does note when heart rate is irregular (occurs every 2-3 months), and gets concerned that it may lead to a heart attack.*

*Discussed associated risk factors, e.g. weight.*

**DIABETES**

Date of Blood Tests: XX/XX/XX; Fasting HbA1c: 7.4% Glucose (Recommended < 7.0): 7.7 mmol/l

[A level of less than 7% is very good, a level between 7% & 8% is adequate, between 8% & 9% suggests the need for improvement, and over 9% is associated with poor control of blood sugar levels]

**Diabetic status:** Determine how long participant has been aware of diabetic status, and current management strategies.

**Education**: The risk of heart disease, stroke and recurrent stroke is much greater in people with diabetes. Control of your glucose levels significantly reduces these risks. Ways to improve your blood sugar levels are:

- A healthy eating plan (e.g. lower total fat intake and find substitutes for saturated fat, reduce sugar intake, and increase consumption of fruit and vegetables)
- Optimizing weight
- Regular physical activity to improve metabolic control
- Medication may be required, but does not substitute for healthy eating and activity
- Encourage participant to discuss diabetes management with GP
- Advise that there is a Diabetes Australia Helpline for further information and support (see contacts card).

**Goal**: Achieve a stable blood glucose level that is below 7 mmol.

**Potential referral:** Determine interest in seeing a dietitian.

**Please document main issues for the participant and advice offered**

*Participant has had diabetes for 30 years, under endocrinologist and with Baker IDI Institute for follow-ups. Participant does blood BSL QID.*

*Participant has good knowledge of medications.*

*Participant does not have diabetic educator, but sees dietician.*

*Participant reads symptoms and recognises if BSL < 4, does get symptomatic.*

*Participant’s GP now monitors and tests urine monthly.*

*We discussed the use of Diabetes App to monitor BSL, foods, and calorie intake.*

**CHOLESTEROL**

Total cholesterol level: 4.1 mmol/l LDL: 2.4 mmol/l HDL: 0.9 mmol/l Triglycerides: 1.7 mmol/l

**Education**: High cholesterol levels are associated with a greater risk of heart disease and stroke recurrence. It is recommended that all people who have had an ischaemic stroke should receive cholesterol lowering therapy. Other ways to help reduce cholesterol levels include increase physical activity, avoid processed food because these have high sources of fat, and increased fruit and vegetable intake. GPs and pharmacists can provide support in these areas.

**Goal**: Maintain a normal cholesterol level, and have an annual cholesterol test.

**Please document main issues for the participant and advice offered**

*Participant has had high cholesterol for over 10 years and has been on medication. Participant has tried dietary changes, does not have fatty foods and uses low fat butter/margarine, occasionally takes cheese, fish and chips, and pizza.*

*Participant’s cholesterol levels have come down since taking medications.*

*Participant also hopes to reduce weight and increase exercise. Participant said “it’s a slow progress”.*

**EXCESSIVE ALCOHOL INTAKE**

The participant drinks once a month or less. On each occasion, the participant takes 1 to 2 drinks

**Education**: Heavy drinking can raise your blood pressure and increase your risk of stroke. It is a good idea to discuss your alcohol intake with your doctor as alcohol may interact with some of your medications or make it harder to control blood pressure.

**Goal**: Limit daily alcohol consumption to 2 standard drinks for men and 1 standard drink for women (show pictures). Everyone should have at least one or two alcohol free days every week.

**If heavy drinker:** Determine whether they may be dependent or not. If dependency is suspected, administer AUDIT questionnaire.

**Interest in cutting down**. Determine whether the patient is interested in cutting down.

**Confidence about succeeding.** Determine how confident the patient is in cutting down. Find out what would be required to increase their confidence in cutting down. Discuss barriers to reducing alcohol. Negotiate and set realistic goals.

**Potential referral:** Determine interest in seeing an alcohol dependency specialist.

**Please document main issues for the participant and advice offered**

*Participant rarely takes alcohol. Participant is aware that heart rate goes up when he does.*

**OVERALL WELL-BEING (Physical Activity, Weight Loss, and Nutrition)**

**Exercise**: the participant:

- Exercises vigorously 0 times/week;
- Walks 30 minutes or more 1 time/week;
- Undertakes moderate-intensity activity 0 times/week.

**Weight** assessed at home: XX/XX/XX; BMI = 35.1 kg/m2; Waist circumference = 126 cm; Waist-Hip Ratio: 1.0

The participant:

- Has gained more than 10kg since his late teens and early twenties;
- Has tried to lose weight 1 time since his late teens and early twenties.

**Nutrition**:

- Choose low fat dairy products;
- Eats 3 serves of vegetables per day (Recommended = 5 serves/day);
- Eats 3 serves of fruit per day (Recommended = 2 serves/day);
- Does not eat pies, pastries, fried foods or takeaway meals more than once a week;
- Does not drink soft drinks, cordials, sport drinks or fruit juice on most days of the week;
- Sodium excretion (Recommended range 40-220): 141 mmol/day
- Adds salt to food at the table;
- Does not add more than 1 teaspoon salt per day at the table.

**Education:** Physical activity, weight, and nutrition all impact on the occurrence of risk factor (e.g. high blood pressure, cholesterol). People who exercise regularly are about 30% less likely to have a stroke and 50% less likely to have cardiovascular disease. Regular exercise can reduce the risk of stroke by lowering blood pressure, assisting in weight loss and reducing cholesterol.

Losing weight improves blood pressure and blood glucose levels.

Diets rich in fruit and vegetables are associated with a lower risk of heart disease and stroke, and reduce the likelihood of developing type 2 diabetes and high blood pressure.

**Activity Goal:** Just 30 minutes per day of moderate-intensity physical activity (either continuous or in bouts of 10-minute intervals) provides health benefits.

**Weight Goal:** Reduce weight if BMI > 25kg/m2

**Nutrition Goals:**

- Include 5 serves of vegetables in your diet every day
- Include 2 serves of fruit in your diet every day
- Avoid eating saturated fats in your diet (e.g. butter, cheese, hidden fats in cakes and pastries)

**Potential referral:** Determine interest in seeing a dietitian.

**Please document main issues for the participant and advice offered**

*Participant has been increasing exercise regime slowly. Participant has treadmill, uses it for 5-10 minutes 4-5 times a week. Participant hopes to increase time on it slowly.*

*Participant has also reduced diet to 2 meals a day. Participant now drinks a lot of soup.*

*Participant has seen a dietician for advice.*

*We discussed the issue of weight gain, participant hopes to decrease weight.*

**MEDICATIONS AFTER STROKE**

The patient is currently taking 10 medications.

**Education:** There are many different medications that your doctor may prescribe to reduce your risk of having another stroke or TIA.

**Goal**: If your doctor prescribes medication, it is important to continue taking it unless the doctor tells you to stop. If you have difficulty in remembering to take your medications then you can try:

- Taking your medication at the same time every day. It is important to get into a routine.
- Using a pill box or dispenser that notes day and times. You can organize this with your local pharmacist.
- Using a medication diary or daily chart to keep track of your medications.

Your doctor will help you to work out the right medication, dosage and timing for your lifestyle. Never stop taking your medication or change how much you take without talking to your doctor. In some cases, suddenly stopping your medication can be dangerous.

If you do not understand any of these things, please discuss with your doctor. Also remember to report any side-effects of your drugs to your doctor. Also remember to tell your doctor about any other medications that you may be taking that were not prescribed by the doctor.

**Encourage patient to talk to their doctor about the medications they are taking.**

**Please document main issues for the participant and advice offered**

*Participant has reduced the amount of Insulin intake since stroke. And it is continually monitored and changed accordingly.*

*Other medications are well tolerated.*

*Participant has a good understanding of the medications being taken and why.*

Please list other family members involved in discussion, and responsibility (e.g. spouse cooking or son smoking).

Interviewed alone. Independent.

**Make appointment with GP for Management Plan in about 1 week (remember to tell receptionist that this will be for a long visit).**
